# Financial Viability and Environmental Sustainability of Fecal Sludge Treatment with Pyrolysis Omni Processors

**DOI:** 10.1021/acsenvironau.2c00022

**Published:** 2022-07-29

**Authors:** Lewis Stetson Rowles, Victoria L. Morgan, Yalin Li, Xinyi Zhang, Shion Watabe, Tyler Stephen, Hannah A. C. Lohman, Derek DeSouza, Jeff Hallowell, Roland D. Cusick, Jeremy S. Guest

**Affiliations:** † Institute for Sustainability, Energy, and Environment, University of Illinois Urbana−Champaign, Urbana, Illinois 61801, United States; § Department of Civil & Environmental Engineering, University of Illinois Urbana−Champaign, Urbana, Illinois 61801, United States; ∥ Biomass Controls PBC, Woodstock, Connecticut 06281, United States

**Keywords:** Omni Processor, fecal sludge management, techno-economic analysis (TEA), life cycle assessment (LCA), sensitivity, uncertainty, resource recovery, carbon sequestration

## Abstract

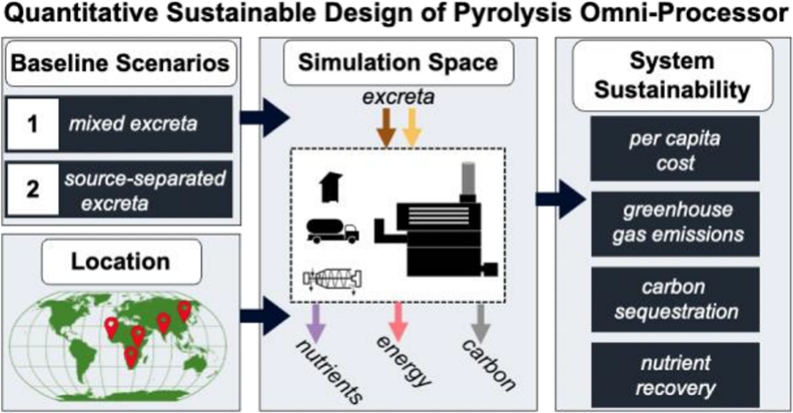

Omni Processors (OPs) are community-scale systems for non-sewered fecal sludge treatment. These systems have demonstrated their capacity to treat excreta from tens of thousands of people using thermal treatment processes (e.g., pyrolysis), but their relative sustainability is unclear. In this study, QSDsan (an open-source Python package) was used to characterize the financial viability and environmental implications of fecal sludge treatment via pyrolysis-based OP technology treating mixed and source-separated human excreta and to elucidate the key drivers of system sustainability. Overall, the daily per capita cost for the treatment of mixed excreta (pit latrines) via the OP was estimated to be 0.05 [0.03–0.08] USD·cap^–1^·d^–1^, while the treatment of source-separated excreta (from urine-diverting dry toilets) was estimated to have a per capita cost of 0.09 [0.08–0.14] USD·cap^–1^·d^–1^. Operation and maintenance of the OP is a critical driver of total per capita cost, whereas the contribution from capital cost of the OP is much lower because it is distributed over a relatively large number of users (i.e., 12,000 people) for the system lifetime (i.e., 20 yr). The total emissions from the source-separated scenario were estimated to be 11 [8.3–23] kg CO_2_ eq·cap^–1^·yr^–1^, compared to 49 [28–77] kg CO_2_ eq·cap^–1^·yr^–1^ for mixed excreta. Both scenarios fall below the estimates of greenhouse gas (GHG) emissions for anaerobic treatment of fecal sludge collected from pit latrines. Source-separation also creates opportunities for resource recovery to offset costs through nutrient recovery and carbon sequestration with biochar production. For example, when carbon is valued at 150 USD·Mg^–1^ of CO_2_, the per capita cost of sanitation can be further reduced by 44 and 40% for the source-separated and mixed excreta scenarios, respectively. Overall, our results demonstrate that pyrolysis-based OP technology can provide low-cost, low-GHG fecal sludge treatment while reducing global sanitation gaps.

## 1 Introduction

A global effort to eliminate sanitation gaps has been galvanized by the United Nations Millennium Development Goals and the more recent Sustainable Development Goals.^1,2^ However, current trajectories suggest that progress will fall short of 2030 targets for universal sanitation coverage and halving the proportion of untreated wastewater.^3−5^ To address the key challenge of safe sanitation for all, traditional interventions typically include centralized treatment with sewered toilets where water is the carrier of human excreta by gravity.^6^ However, the implementation of such solutions require large capital investments, water for conveyance, and maintenance of expansive pipeline networks.^6^ Alternatively, pit latrines are a common sanitation technology used by over 1.5 billion people globally,^6,7^ and container-based sanitation systems (i.e., waterless toilets that capture excreta) have recently gained traction due to their ability to safely collect excreta.^8−10^ Although pit latrines and container-based sanitation systems tend to be less expensive and easier to implement relative to sanitary sewers, they require routine emptying as containment of excreta is only part of the sanitation value chain (user interface, collection, emptying and conveyance, treatment, and reuse).^6,11^ Community-wide deployment of container-based sanitation systems creates greater needs for collection, treatment, and reuse at large scales. Thus, an opportunity exists for innovative solutions to be developed for non-sewered fecal sludge management at large scales, which could significantly impact on global sanitation needs.^12^

Thermal treatment systems (e.g., pyrolysis, gasification, combustion) represent one potential pathway for non-sewered fecal sludge (i.e., excreta that contains solids) treatment at large scales. These systems leverage the caloric value of feces (upward of 25.7 MJ·kg^–1^ dry basis) to reduce fecal sludge volume, destroy pathogenic organisms, and remove many harmful chemical compounds.^13,14^ The relatively high treatment temperature of pyrolysis (e.g., 350–800 °C) transforms feedstock (e.g., fecal sludge) into biochar, a graphitic solid that can be used to enhance agriculture soil properties and qualifies for carbon sequestration credits.^15,16^ Biochar can also be used to produce briquettes that can be used as a fuel for cooking or heating.^17^ Additionally, the produced thermal energy from pyrolysis can be converted to electrical energy or leveraged to dry influent fecal sludge.^18,19^ While the moisture content of fecal sludge may be considered an obstacle to thermal treatment, dewatering followed by drying with the produced thermal energy can achieve the minimum requirements in many cases.^13,20^

The development of thermal treatment systems has been accelerated through the Bill & Melinda Gates Foundation’s Reinvent the Toilet initiative.^21,22^ Community-scale, non-sewered sanitation systems developed through this program have been coined Omni Processors (OPs).^21,22^ From a technological standpoint, these community-scale fecal sludge management systems are marketed as optimized sludge treatment technologies leveraging thermal treatment to inactivate pathogens and recover energy from bodily waste.^22^ These systems are proposed to be equipped with remote monitoring and have limited requirements for on-site operators.^23−25^ Additionally, design teams note that OPs can handle a variety of inputs (e.g., menstrual hygiene materials, municipal solid waste, and organic wastes),^26^ which can cause blockages in sewage collection systems and interfere with the performance of other fecal sludge management processes.^27,28^ Undoubtedly, addressing sanitation goals through technology deployment should consider the critical challenges of stakeholder engagement and social acceptability;^10,29−33^ nonetheless, costs, energy, and life cycle environmental impacts are three indicators that are potentially of urgent relevance to decision-makers.

Despite the efficacy of thermal treatment being well studied, research on the relative sustainability of novel OPs is limited. A report from 2012 explored the general themes of OPs and the types of treatment processes that these systems could leverage.^34^ However, this early-stage study highlighted biological processes (e.g., anaerobic digestion) as a core treatment strategy and argued that thermal conversion (e.g., pyrolysis) was too complex with no systems available for fecal sludge management at the time.^34^ Although biological treatment is common for community-scale treatment, greenhouse gas emissions (i.e., CH_4_ and N_2_O) can be substantial, as deviation from optimal operation conditions commonly occurs.^35,36^ Significant innovation has made thermal treatment of excreta feasible, with several of the OPs having gone through multi-year pilot studies, and limited publicly available information suggests promising technical viability.^22,25,26,37−40^ For example, a laboratory study of the Biogenic Refinery (a pyrolysis-OP from Biomass Controls PBC) showed that this system could support its steady state electrical and heating needs when paired with a combined heat and power system (i.e., it does not require any energy inputs).^41^ The development of OPs as part of a portfolio of technologies to address global sanitation needs presents a timely opportunity to investigate their relative sustainability.

The objectives of this work were (i) to characterize the financial viability and environmental implications of fecal sludge management via pyrolysis-based OP technology and (ii) to elucidate the key drivers of system sustainability. To this end, we gather data and leverage quantitative sustainable design (QSD) to characterize the relative sustainability of the Biogenic Refinery 4018 (Biomass Controls PBC). Performance of this pyrolysis-OP was evaluated through quantitative models that leverage both pilot and full-scale data over extended operation times (several years). Two different implementation scenarios with different frontend facilities (pit latrines and container-based sanitation) provide the baseline for this study. By leveraging an open-source QSD tool (QSDsan^42^), trade-offs between these scenarios were assessed across the simulation space spanning the feasibility ranges of various design decisions and technological parameters. Outcomes were evaluated across contexts by altering key assumptions to simulate system deployment in five countries of interest (China, India, Senegal, South Africa, and Uganda). Key drivers of system sustainability were identified through uncertainty (Monte Carlo simulation) and sensitivity (Spearman’s rank correlation coefficients) analyses. Lastly, carbon and nutrient balances for each scenario were evaluated to quantify the potential of these resources to offset sanitation deployment costs through carbon sequestration credits and fertilizer sales.

## 2 Methods

### 2.1 System Overview and Scenarios

To characterize the relative sustainability of the Biogenic Refinery 4018 (Biomass Controls PBC), we consider two baseline scenarios (Figure S1). Baseline scenario 1 models the treatment of a mixed excreta stream from pit latrines where dewatering by screw press is required before solids are treated with the OP. The pit latrines in this analysis are assumed to be dry (i.e., do not require pour or mechanical flushing) to be consistent with the moisture content in the pilot-scale deployment of this system. Baseline scenario 2 includes urine-diverting dry toilets where source-separated feces and urine are collected and processed independently. Feces is broken down by a grinder before being used as feedstock for the OP. Separately, urine is processed to recover nutrients for fertilizer through struvite precipitation and ion exchange.^43,44^ The refinery has three main assemblies: (i) a carbonizer base, (ii) a pollution control device, and (iii) heat exchangers. The carbonizer base is the central location for the combined pyrolysis and combustion process. The feedstock is placed into the carbonizer base by an auger and exposed to a high-temperature, low-oxygen environment where the volatile gases are released and subsequently combusted to generate thermal energy. The generated syngas from pyrolysis is passed through a catalytic converter within the pollution control device to reduce emissions of remaining pollutants and improve thermal efficiency before proceeding to the heat exchanger for thermal energy recovery. In these scenarios, thermal energy within the refinery is utilized for generating electrical energy with the oil heat exchanger and then drying of feedstock with the hydronic heat exchanger.

Design, simulation, sustainability characterization, and uncertainty and sensitivity analyses of the OP systems were performed in Python (version 3.8)^45^ using QSDsan (an open-source, community-led platform for quantitative sustainable design of sanitation and resource recovery systems).^42^ The code is openly available on Github.^46^ In this parallel analysis, we generate estimates of per capita costs for the Biogenic Refinery (12,000 users·d^–1^), along with associated environmental impacts assuming a production scale of 10,000 units. We assumed a lifetime of 20 yr for the OP with replacement of individual parts based on their lifetimes and fixed performance data provided by the design team. The system includes installation of the technologies, on-site construction, frontend (toilet and onsite storage), and pretreatment requirements. Generally, a ±10 to 25% variation was applied to assumed values (depending on data availability).

### 2.2 Economic Analysis

We used discounted cash flow analysis to calculate daily per capita cost based on capital, operation and maintenance, and electricity expenses (separate from operation). The expenses or revenue was amortized. Specifically, initial capital costs were distributed over the lifetime of the system, with a discount rate adjusting for the diminishing value of money over time. For capital costs of OP, a learning curve equation was used to conservatively estimate costs at scale to produce 10,000 units.^47,48^ Operation and maintenance costs were estimated for consumables and replacement parts, and labor costs were estimated based on operator wages and hours of labor. Electricity requirements were estimated based on the energy needs of each unit within the OP and typical electricity costs per kilowatt-hour. The total cost was adjusted to account for income tax obligations (at a tax rate of 20–35%). The objective of this analysis is to estimate the daily user fee necessary to account for the full costs of the system (e.g., meeting annual operating expenses while accounting for initial capital requirements). Details on the modeling procedures for costs are described in the Supporting Information (Section S2).

### 2.3 Environmental Analysis

Environmental impacts were estimated from four sources: capital, energy, direct impacts from excreta, and operation and maintenance. This analysis focused on the life-cycle global warming potential (i.e., GHG emissions as kg-CO_2_ equivalents). Impacts were estimated from the system’s construction materials and electricity demands using the ecoinvent v3.6 database^49^ and the U.S. EPA’s Tool for the Reduction and Assessment of Chemicals and Other Environmental Impacts, TRACI 2.1 v1.03.^50^ Direct GHG emissions from excreta during treatment were estimated using assumptions related to typical excretion of carbon and nitrogen^51−54^ and treatment conditions.^6,55−67^ The total impacts of fecal sludge management were normalized to a per capita basis over the course of a year (i.e., kg CO_2_ eq·cap^–1^·yr^–1^). Details on the modeling procedures for environmental analysis are described in the Supporting Information(Section S2 ).

### 2.4 Key Assumptions Assessed across Scenarios

#### 2.4.1 Decision Variables

For the two baseline scenarios, different excreta collection methods were included (i.e., pit latrines and container-based sanitation). A wide breadth of emptying periods for the pit latrines were initially assumed (a triangular probability distribution with a minimum of 0.3 yr, maximum of 2.4 yr, and mode of 0.8 yr), and power law regression was used to estimate the emptying fee associated with a given sludge volume.^30^ Emptying period and other assumptions related to pit latrines were treated as independent, uncertain variables (shown in Tables S1 and S2) to assess which ones had the largest influence on the uncertainty of sustainability indicators. Further analysis was completed to assess the impact of emptying period (frequent as 0.5–1.0 yr and infrequent as 2.0–2.5 yr) as well as emptying costs. The collected excreta from the latrines were assumed to be transported to the OP via truck. Additional details on emptying and conveyance are included in the SI Section S4 and Tables S1 and S2. For the other baseline scenario where container-based sanitation facilities were used, source-separated urine and feces were stored in removable containers that were collected frequently (e.g., twice per week) by dedicated employees. Since these conveyance systems are more complex and require more maintenance, the capital and maintenance costs were higher than those from latrines.^30^ Containers were assumed to be collected by pushcarts from individual toilets and then transported to the central treatment facility by truck. Urine from containers was processed by ion exchange and struvite precipitation to recover nutrients for fertilizer, following previously published assumptions for efficiencies, costs, and consumables.^43,44^ The potential costs and emission offsets from resource recovery were assessed for both scenarios. The biochar products were assumed to be pathogen-free due to the high treatment temperature. Nutrients from ion exchange and struvite precipitation were assumed to offset emissions from fertilizer production and were given a discounted economic value (baseline of 25%) from current fertilizers.^30,44^

#### 2.4.2 Technological Parameters

For the Biogenic Refinery, pretreatment by dewatering is necessary when feedstock has a moisture content greater than 85% (e.g., latrine sludge). For dewatering by screw press, information was compiled from Biomass Controls’ pilot studies and the manufacturer FAN (a company of the Bauer Group). To increase solids removal, a cationic polymer is added prior to feedstock entering the screw press, and this treatment process is assumed to reduce the moisture content to 65%. Information on costing, energy, and materials for the screw press was collected from literature^51^ and various suppliers. According to the manufacturer, the Biogenic Refinery can process feedstock at a rate of 18 kg·h^–1^ (moisture content of 35%). We used empirical models for the Biogenic Refinery based on information from the design team, their pilot systems, and a laboratory study of the system.^41^ These included assumptions that the pyrolysis process liberates embedded energy from fecal sludge for drying and generation of electrical energy. The dryer is assumed to provide the necessary heat to reduce the moisture content from feedstock from 85 to 35%. The oil heat exchanger with combined heat and power is assumed to produce an average of 1.65 kW when the OP is running. The estimates for N_2_O emissions in pyrolysis were updated based on precursors to atmospheric formation of N_2_O (i.e., NH_3_ up to 4% of total N and HNCO [fulminic acid formed during pyrolysis] up to 10% of total N; Table S1).^68,69^

#### 2.4.3 Contextual Parameters

To explore how economic and environmental outcomes might change across different contexts, general assumptions were changed to those that reflect representative conditions in specific countries. Specifically, we used country-specific data on electricity prices,^70^ electricity mixes (to estimate GHG emissions associated with energy requirements),^71^ calorie and protein intake (to estimate chemical oxygen demand (COD) and N excretion, leading to direct emissions of CH_4_ and N_2_O),^72,73^ and labor wage rates (for construction and operation and maintenance labor).^74^ With regard to all of these country-specific inputs, we collected data for five countries of interest: China, India, Senegal, South Africa, and Uganda. However, labor wage rates were not available for India and Senegal, and in these cases, we used average values calculated from wages in the other three countries. Results of this analysis offer insight into how local conditions could affect outcomes when deploying the OP across a range of contexts.

### 2.5 Uncertainty and Sensitivity Analyses

A critical aspect of the QSD methodology involves the incorporation of uncertainty. For each uncertain parameter (290 parameters for the mixed excreta and 319 parameters for the source-separated excreta), distributions are defined (see appended spreadsheets for all parameters) and an additional variability of up to 25% is added to each unit cost and environmental impact factor. This variability was added to account for factors such as the spatial heterogeneity of material prices and impacts. For all scenarios, Monte Carlo simulation with Latin hypercube sampling (10,000 samples) was used to include uncertainty.^75^ This process produced a distribution of results for which the median, 5th percentile, and 95th percentile values from the uncertainty analysis are shown in the results. The input and output distributions from the simulations also were used to calculate Spearman’s rank correlation coefficients as a measure of the results’ sensitivity to individual parameters. In this context, sensitivity refers to the degree to which an output (i.e., costs and GHG emissions) correlates with a single input parameter. Spearman’s coefficients are calculated by ranking the values in each input and output distribution (e.g., the lowest value is assigned a rank of 1, the second lowest is assigned a rank of 2, and so on) and determining the correlation between these ranks. This correlation is shown by a coefficient value that represents the degree to which an arbitrary monotonic function can describe the relationship between the input parameter and output value. Coefficient values range from −1 and 1, with a larger absolute value signifying a stronger correlation. For this work, absolute values of coefficients are shown in the results.

## 3 Results and Discussion

### 3.1 Financial Viability and Environmental Implications of Omni Processor Technology

**Figure 1:**
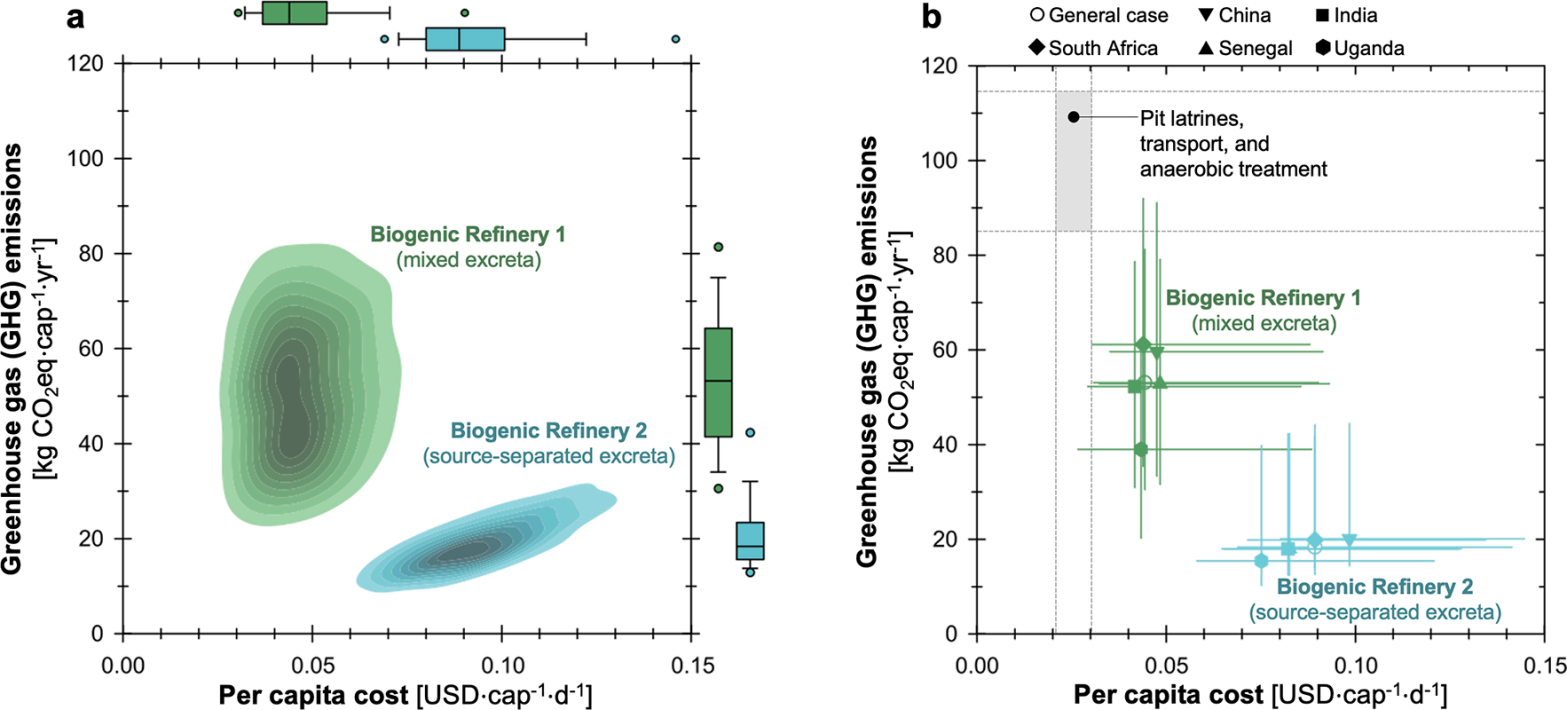
Estimates of economic and environmental outcomes associated with the Biogenic Refinery under (a) two different bodily waste management scenarios and (b) different deployment contexts (the general case as well as five countries of interest). The kernel density maps represent 10,000 Monte Carlo simulations. The horizontal position corresponds to per capita cost, and the vertical position corresponds to GHG emissions. Box and whisker plots along the axes represent the scenario-specific distribution of GHG emissions and daily per capita cost on the vertical and horizontal axes, respectively. This plot shows the results from the Biogenic Refinery under two scenarios: mixed excreta stream with pit latrines (green) and source-separated excreta with urine-diverting dry toilets (blue). The box and whisker plots represent the median values (center line), 25th and 75th percentiles (bottom and top of box), 10th and 90th percentiles (lower and upper whiskers), and 5th and 95th percentiles (points on either end of the whiskers).

Since the deployment of this optimized system is a priority, our analysis primarily focuses on exploring the context in which the system is used. For the Biogenic Refinery, the treatment of mixed excreta is shown to have a lower cost with higher GHG emissions than the treatment of the source-separated excreta (Figure 1a). Specifically, the daily per capita cost in the mixed excreta scenario is estimated to be 0.05 USD·cap^–1^·d^–1^ (median) with a range of 0.03–0.09 USD·cap^–1^·d^–1^ [hereinafter, 5th–95th percentiles are shown in brackets]. On the other hand, the source-separated scenario is estimated to have a per capita cost of 0.09 [0.07–0.14] USD·cap^–1^·d^–1^. The higher costs of the source-separated scenario are accompanied by significantly less GHG emissions over the system lifetime. The emissions from the source-separated scenario were estimated to be 18 [12–41] kg CO_2_ eq·cap^–1^·yr^–1^, compared to 53 [30–81] kg CO_2_ eq·cap^–1^·yr^–1^ for the mixed excreta. The trade-offs between cost and GHG for these scenarios present varying opportunities for the deployment of this OP, where the mixed excreta scenario offers a lower per capita cost option but with higher GHG emissions.

### 3.2 Performance of the Omni Processor across Contexts

To assess how application context may affect the economic and environmental outcomes associated with the OP, we compared our general estimates with results calculated for five specific countries (i.e., China, India, South Africa, Senegal, and Uganda). A wider variation in emissions across contexts is observed for the treatment of mixed excreta, while cost varies more for the source-separated scenario across contexts (Figure 1b). These observations can primarily be attributed to local diet. For the mix stream treatment, countries with higher calorie intake (i.e., China and South Africa) excrete waste with higher carbon content (i.e., higher COD). Pit latrines have higher emissions when the excreta stream is COD-rich. On the other hand, countries with lower calorie intake (e.g., Uganda) have lower emissions than the baseline during mix stream treatment. For the source-separated scenario, country-specific deviations for emissions are minimal due to the relatively low direct fugitive emissions from the more frequently emptied containers for collection. The observed variation in cost for the source-separated scenario can be attributed to greater protein intake resulting in higher chemical input requirements for nutrient recovery (i.e., ammonia from ion exchange). However, this country-specific analysis does not include monetary value for the recovered resources, which would help to offset these costs (explored below). Overall, the estimates for emissions of this scenario had overlapping distributions, suggesting that GHG emissions of the treatment of source-separated excreta are relatively independent of context. Generally, it should be noted that the country-specific assumptions do not cover the full set of conditions that may affect performance, and the country-specific averages that we used do not capture (potentially large) sub-national variations.

Both scenarios fall below the estimates of GHG emissions for anaerobic treatment of fecal sludge collected from pit latrines (gray region in Figure 1b). Conversely, the costs of both scenarios are greater than this benchmark system. For this analysis, estimates for pit latrines and transportation were adopted from the baseline treatment of mixed excreta (which also used pit latrines), while estimates for anaerobic treatment followed previous analyses.^10^ A wide variation in emissions (85–115 kg CO_2_ eq·cap^–1^·yr^–1^) and costs (2–3 USD·cap^–1^·d^–1^) from this benchmark sanitation system was estimated. This variation is due to the broad assumptions that were used, which can greatly impact costs (e.g., emptying fee and period and capital costs) as well as emissions (e.g., emptying period and decay assumptions). The construction, operating conditions, and performance of pit latrines can greatly vary across contexts.^6^ Regardless of the broader variation in these results, OP in both scenarios has lower emissions at a higher cost.

### 3.3 Elucidating Drivers for Cost and Emissions

The next step in our analysis was to elucidate drivers for cost and GHG emissions. First, the overall system was broken down into individual units, and the relative contributions to cost and emissions were estimated. The categories for cost included capital, operation and maintenance, and electricity. The categories for emissions included capital, energy, operation and maintenance, and direct emissions from waste. The results from this analysis are shown in Figure S2, where the percentage of total daily per capita cost and percent of annual GHG emissions per user are demonstrated for both scenarios. Additionally, the total magnitude of daily per capita cost and annual GHG emissions per user are shown in Figure S3.

For the mixed excreta scenario, the largest median costs were attributed to the capital costs of the pit latrines (31% [19–52%] of total per capita cost) followed by the electricity requirements for pretreatment (i.e., screw press) of the fecal sludge (16% [6–34%]). The next highest contributors to the total cost of the OP were those related to operation and maintenance, including pit latrine emptying (12% [5–22%]), full-time operators of the OP (16% [8–25%]), and transport of the fecal sludge from the pit latrines to the OP system (15% [10–23%]). The costs related to the operation and maintenance of the carbonizer base (i.e., the unit of the OP that is responsible for the pyrolysis) was 2.4% [1–5%], due to the high frequency that parts need to be replaced in this unit. It is notable that energy production of the combined heat and power system was able to offset total costs (−1% [−2 to −0.05%]) by producing energy (i.e., negative cost). The remaining relative contributions were relatively small (<2% on average). The direct emissions from waste from the pit latrine dominated the GHG emissions for the mix excreta scenario (64% [39–78%]). The GHG emissions from the energy required to support pretreatment by dewatering accounted for 16% [5–41%] of the total emissions. The third highest contributor to GHG emissions was capital emissions from the pit latrine (14% [6–35%]). The remaining contributors to GHG emissions were relatively small (each had a median contribution of <2%).

For the treatment of source-separated excreta, the largest median costs were attributed to transport (34% [21–46%]) due to the high frequency of container collection and transport to the OP (every 1–9 d with triangular distribution, mode of 3.5). The second greatest costs were the operation and maintenance expenses from the treatment of the liquid stream (23% [14–33%]). The liquid treatment included the units for recovery of struvite via precipitation and ammonium sulfate via ion exchange, both of which require consumables. The next highest contributors to costs for this scenario were the urine-diverting dry toilets’ capital (17% [10–36%]) and operation and maintenance (10% [5–21%]). The other notable costs were for operators (8% [5–12%]) and operating and maintenance costs of the carbonizer base (2% [1–3%]). Since excreta is separated at the source and processed independently, only a grinder is required for pretreatment, which has significantly lower energy requirements than the dewatering pretreatment necessary in the mixed excreta scenario. The construction emissions (i.e., capital) of the urine-diverting dry toilets were the most significant contributor to GHG emissions for this scenario (55% [38–80%]) due to the large number of bricks that are required to house the containers of the urine-diverting dry toilets.^10,44^ The proceeding contributors to GHG emissions were the operation and maintenance of the liquid treatment (23% [10–35%]), energy for pretreatment (7% [3–15%]), and transportation emissions (5% [1–15%]). The remaining contributors to GHG emissions from the treatment of source-separated excreta were relatively small (<1% on average).

These findings present the specific trade-offs in deployment that need to be considered beyond only the magnitude of costs and GHG emissions. For both scenarios, it is observed that the operation and maintenance of the OP is a critical driver to total per capita cost, whereas the contribution from capital cost of the OP is much lower because it distributed over a relatively large number of users (i.e., 12,000 people) over the system lifetime (i.e., 20 yr). Parameters that are major contributors to the total costs or emissions are those that do not benefit from economies of scale or are not distributed over the number of users (e.g., the frontend, transport, and operation and maintenance of the OP). The necessary costs related to transport and operation can be viewed as beneficial since these create jobs that can stimulate the local economy. Although the pit latrines have highly variable operating costs and GHG emissions, they are commonly used in many parts of the world and may already have existing infrastructure for emptying;^6,11^ thus, the OP could be integrated without the need to widespread frontend construction in such contexts. However, the energy requirements for pretreatment by dewatering could be problematic for sustained operation in locations with frequent electricity blackouts.^76,77^ For the treatment of source-separated excreta, the greater emptying requirements for the urine-diverting dry toilets and materials necessary for liquid treatment may not always be feasible, particularly in remote contexts. The characterization of these trade-offs helps navigate decision-making in the deployment of OP technology and highlight key areas of potential improvement in its sustainability.

**Figure 2:**
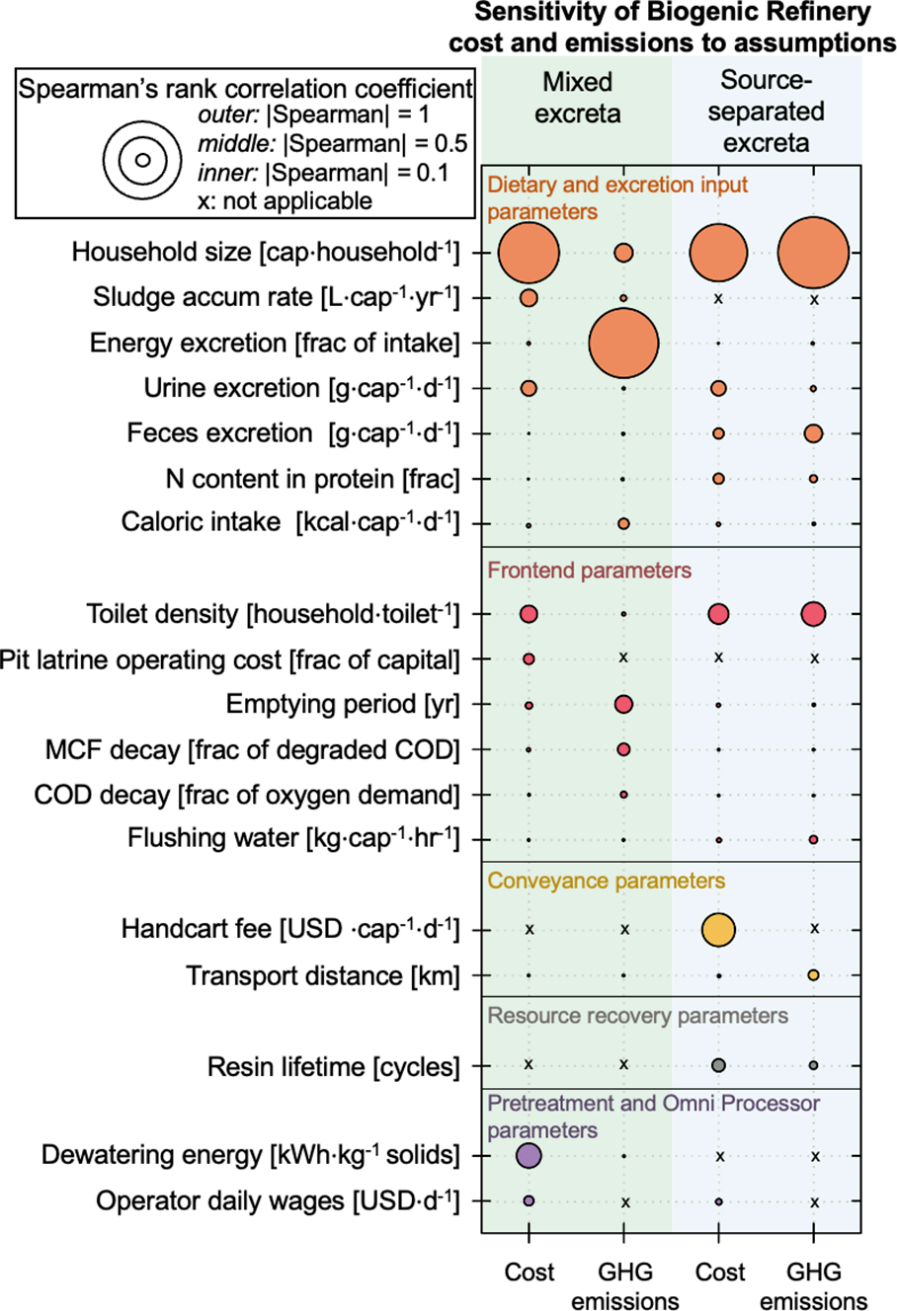
Spearman’s rank correlation coefficients for the daily per capita cost and greenhouse gas (GHG) emissions for the Biogenic Refinery. Parameters are divided into categories along the sanitation chain.

To reveal which parameters and assumptions from our analysis influenced the outcomes of cost and GHG emissions, we conducted a sensitivity analysis. Our uncertainty analysis for the Biogenic Refinery included 290 parameters for the mixed excreta and 319 parameters for the source-separated excreta. These results are separated into different categories along the sanitation chain (Figure 2). In both scenarios, cost and GHG emissions were found to be highly sensitive to household size and toilet density, which in combination determined the number of users per toilet. Our range for the number of users per toilet was generally 3–35 (a median of 4 people per household with a standard deviation of 1.8 and 3–5 households per toilet) and was based on survey results of an urban informal settlement.^10^ The impact of this estimate on deployment of the OP suggests aggregated household toilets may be the most cost effective or low-emissions practice. For example, this practice may include toilets from individual households connected to a central holding tank within a neighborhood or apartment building as well as deployment for public sanitation access (e.g., schools, parks, and informal settlements). It is important to note that this practice may only be viable for pour-flush and mechanical-flush pit latrines (with flowing sludge). For the mixed excreta scenario, the sludge accumulation rate was found to influence both cost and GHG emissions; energy excretion was found to be a key source of uncertainty GHG emissions since it influences the amount of COD in excreta (thus, the amount of fugitive CH_4_ and N_2_O from COD). Other assumptions related to excretion were found to have a relatively higher influence on costs of the source-separated scenario since these will affect how much consumables are needed for resource recovery units (i.e., struvite precipitation and ion exchange). The impacts of the parameters related to excretion support the context-specific findings, where calorie intake impacts emissions from the mixed treatment scenarios and protein intake impacts cost from the source-separated scenarios (as described in context-specific analysis). For assumptions relating to the frontend, the GHG emissions from the mixed excreta scenario were sensitive to the emptying period of the pit latrine, while outcomes for the source-separated scenario were found to be sensitive to conveyance and resource recovery scenarios. The influences of these parameters on costs and emissions are further explored in Figure S4 (described below). Significant parameters related to pretreatment and the OP included operator wages for both scenarios and dewatering energy for the treatment of mixed excreta. Our general range for wages was 14.55–43.68 USD·d^–1^. This value can vary greatly across contexts and should be considered in deployment. Overall, identifying which parameters are most influential to cost and emission uncertainty provides a basis for improving the relative sustainability of the OP.

### 3.4 Improving Relative Sustainability of a Community-Integrated OP

**Figure 3:**
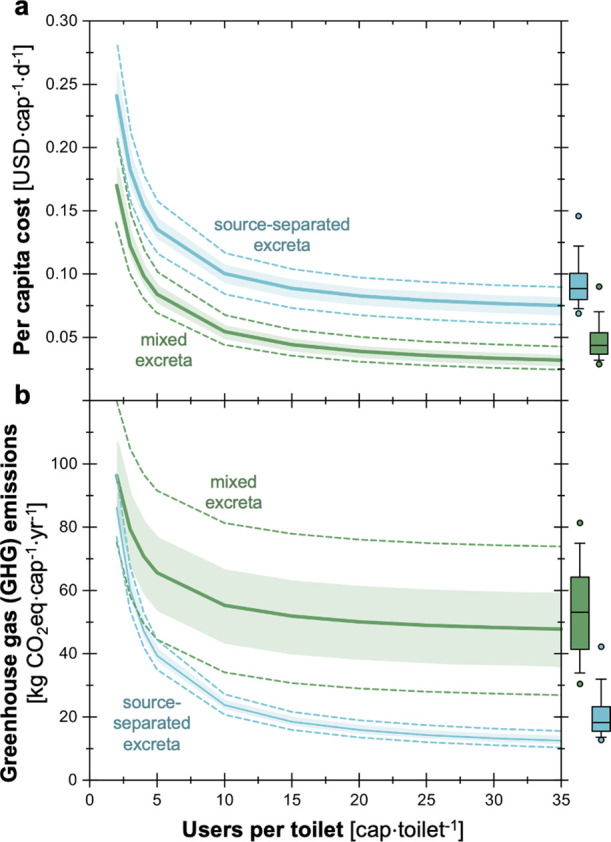
Impact of the number of users per toilet on (a) daily per capita cost and (b) greenhouse gas (GHG) emissions. This analysis was performed holding all other parameters constant. The box and whisker plots show original estimates with the median values (center line), 25th and 75th percentiles (bottom and top of box), 10th and 90th percentiles (lower and upper whiskers), and 5th and 5th percentiles (points on either end of the whiskers) from the uncertainty analysis with 1000 Monte Carlo simulations. The original assumption for the number of users per toilet was generally 3–35 (a median of 4 people per household with a standard deviation of 1.8 and 3–5 households per toilet).

To explore avenues for improving the sustainability of the OP, we varied the estimates of several parameters that were identified in the sensitivity analysis. The assumption of the number of users per toilet will impact the number of toilets necessary to support populations that excreta treated by the OP (i.e., 12,000 people). In our models, this assumption will influence waste collection as well as toilet construction and operation and maintenance. To investigate the impact of the number of users per toilet on per capita cost and GHG emissions per user, we varied this parameter from 1 to 35 users per toilet (Figure 3). At the extreme case of 1 user per toilet (i.e., all 12,000 people have their own toilet), the total per capita costs were 0.31 [0.25–0.38] USD·cap^–1^·d^–1^ for the mixed excreta and 0.41 [0.36–0.49] USD·cap^–1^·d^–1^ for the source-separated excreta (Figure 3a). These estimates of per capita cost drastically decrease until approximately 10 users per toilet to 0.06 [0.04–0.07] USD·cap^–1^·d^–1^ for the mixed excreta and 0.10 [0.08–0.12] USD·cap^–1^·d^–1^ for the source separated excreta. At the extreme case of 35 users per toilet, the costs for both scenarios plateau to 0.03 [0.02–0.04] USD·cap^–1^·d^–1^ for the mixed excreta and 0.08 [0.07–0.09] USD·cap^–1^·d^–1^ for the source-separated excreta. Similar trends were observed for the GHG emissions as a function of the number of users per toilet; however, the reduction in GHG emissions for an increased number of users is not as drastic (Figure 3b). Specifically, at 1 user per toilet, the total GHG emissions were 148 [124–173] kg CO_2_ eq·cap^–1^·yr^–1^ for the mixed excreta and 163 [145–184] kg CO_2_ eq·cap^–1^·yr^–1^ for the source-separated excreta. Here, the capital intensive urine-diverting dry toilets have higher emissions than the direct emissions intensive pit latrines. While at 10 users per toilet, the total GHG emissions were 55 [34–81] kg CO_2_ eq·cap^–1^·yr^–1^ for the mixed excreta and 23 [21–27] kg CO_2_ eq·cap^–1^·yr^–1^ for the source-separated excreta. For the mixed excreta, the already high and uncertain direct emissions from pit latrines do not efficiently scale with the number of users. On the other hand, since urine-diverting dry toilets have low direct emissions (from frequent emptying), their GHG emissions (primarily capital) can scale with the increased number of users per toilet.

Since the number of users per toilet may be not controllable in all contexts, we explored the impact of varying several other parameters that could be controlled. This analysis included varying the impact of the number of households per toilet (with an uncertain number of people in each household), as well as other parameters that were identified to be significant from the sensitivity analysis (Figure S4a). Pit latrine emptying fees and number of households per toilet were found to influence per capita cost (Figure S4a), where lower emptying fees and a greater number of households per toilet yielded the lowest cost (i.e., 0.03 [0.02–0.06] USD·cap^–1^·d^–1^ without an emptying fee and six households per toilet). For GHG emissions from the mixed excreta scenario, the latrine emptying period was set to representative ranges of short (0.25–0.5 yr) and long (2.0–2.5 yr) periods between emptying (Figure S4b). Frequent emptying generated an average of 30% less total GHG emissions than infrequent emptying over the range of households per toilet while having a minor impact on total per capita cost. Thus, for the deployment of the Biogenic Refinery, frequent emptying would be preferred over infrequent, when possible. Locations with pit latrines in proximity to one another or near central holding tanks may present opportunities for more frequent emptying. For the source-separated excreta, efficiencies of resource recovery (i.e., resin lifetime for ion exchange, adsorption density for ion exchange, and filter reuse for struvite precipitation) were found to directly influence costs and GHG emissions (Figure S4c,d). These results reveal specific parameters that need to be considered and evaluated for deployment to ensure the overall sustainability of the OP.

An additional avenue to integrate OPs with communities can be to treat other organic waste streams at the same time as fecal sludge. For example, the disposal of food waste or agricultural residues can be an environmental challenge in some communities.^78,79^ The cost associated with including agricultural residue as a feedstock to pyrolysis was assessed for the treatment of mixed excreta with 10,000 users and 2000 user equivalents (in terms of mass loading) of rice husks. Thus, the mass loading to the pyrolysis unit was equivalent for the (i) 12,000 user and (ii) 10,000 user plus agricultural residue scenarios (Section S3). When the residue was assumed to be free (i.e., 0 USD·kg^–1^), the per capita cost of the system was estimated to be 0.05 [0.04–0.09] USD·cap^–1^·d^–1^ (Figure S5). When the price of the agricultural residue was set to be 0.25 USD·kg^–1^, daily per capita cost increased less than 3% on average. Overall, these costs are similar to the mixed excreta 12,000-user scenario (without agricultural residue; Figure 1), suggesting that excreta can be supplemented with residues without driving up costs. Adding agricultural residues may have operations benefits as well. For instance, Biomass Controls has added dry biomass to reduce the moisture content of fecal sludge. Finally, the inclusion of agricultural residues with fecal sludge has been shown to increase nutrients and fixed carbon concentrations of produced biochar.^80^

### 3.5 Charting a Pathway for Research, Development, and Deployment

**Figure 4:**
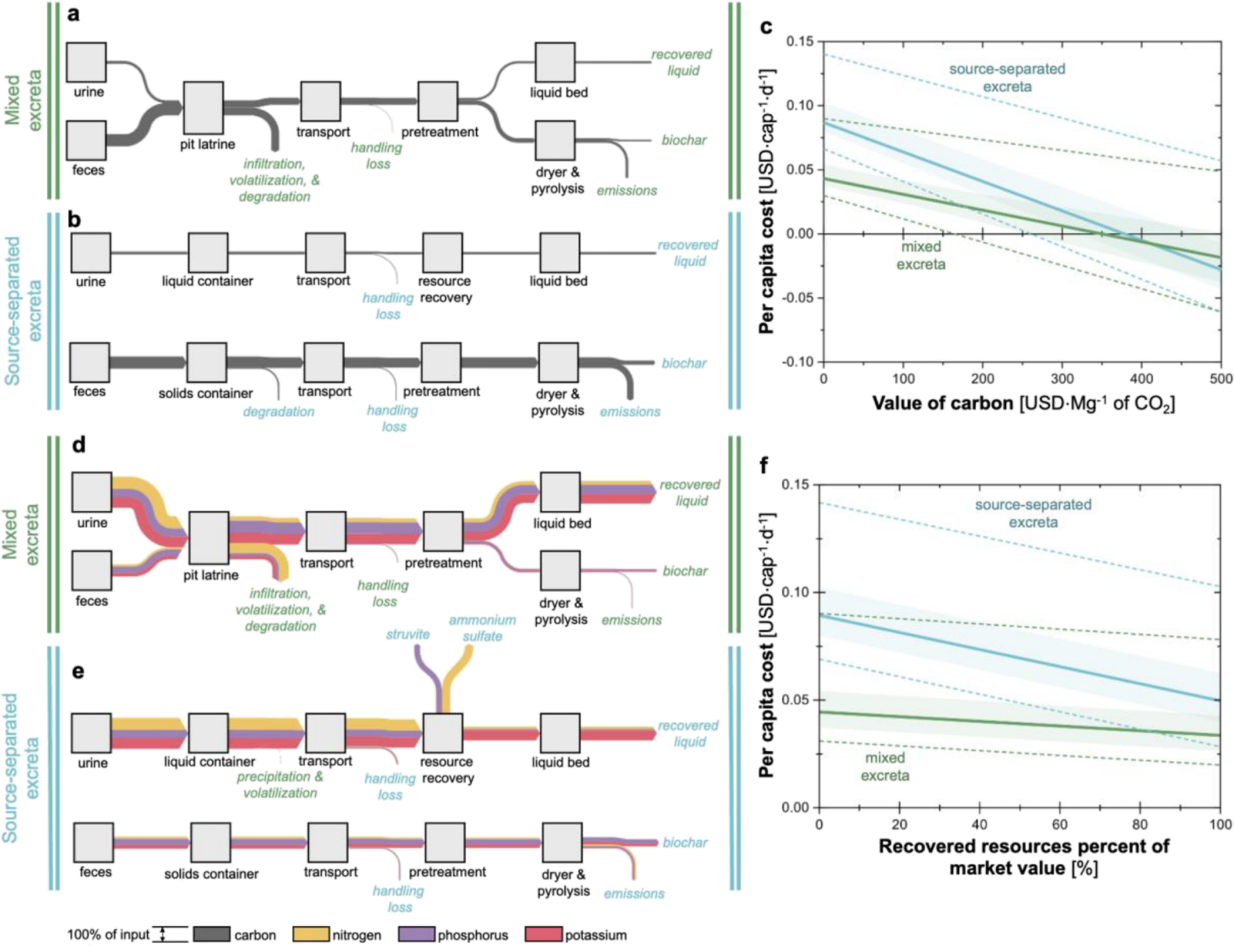
Carbon recovery potential for treatment of (a) mixed excreta and (b) source-separated excreta. (c) The impact of the value of carbon credits on per capita cost for both scenarios using pit latrines, transport, and anaerobic treatment as a comparison for carbon reduction. Nitrogen, phosphorus, and potassium recovery potentials for treatment of (d) mixed excreta and (e) source-separated excreta. (f) The impact of the value of recovered resources with increased percent of market value on per capita cost is shown for both scenarios. The Sankey diagrams show median flows and losses of carbon through each stage of the sanitation chain. All flows are shown relative to 100% of initial inputs.

In the final stage of our analysis, we tracked carbon, nitrogen, phosphorus, and potassium through each of the scenarios (Figure 4). The mixed excreta system has comparably low capture of carbon as biochar (12% [3–38%]), due to the carbon emissions associated with pit latrines (Figure 4a). Source separation reduces the loss of carbon in the frontend and captures more carbon as biochar for the source-separated scenario (i.e., 28% [10–81%] of carbon is captured as biochar; Figure 4b).

To understand the opportunities for carbon sequestration via biochar, we evaluated the per capita cost as a function of carbon sequestration credits (i.e., value of carbon) when the Biogenic Refinery would be implemented in place of a more common sanitation system. For this reference system, we estimated the GHG emissions from a sanitation system consisting of pit latrines, transportation of fecal sludge to a centralized treatment facility, and anaerobic treatment (the benchmark system shown in Figure 1). Holding all equivalent parameters consistent between the technologies, we calculated the per capita cost of the system by including the value of mitigating carbon emissions (Figure 4c). Although the current carbon market is fragmented, estimates suggest that an average value of 34–64 USD·Mg^–1^ of CO_2_ by 2025 can set the course for a 2050 net-zero CO_2_ emission target.^81^ Biochar, a relatively stable carbon product, can fetch on average 181 [111–686] USD·Mg^–1^ of CO_2_ on current markets.^16^ At 150 USD·Mg^–1^ of CO_2_, the per capita cost of the mixed excreta scenario was 0.03 [0.01–0.08] USD·cap^–1^·d^–1^ and the source-separated excreta scenario was 0.05 [0.03–0.11] USD·cap^–1^·d^–1^. The per capita costs are entirely offset by carbon at an average cost of 351 USD·Mg^–1^ of CO_2_ for the mixed excreta and 380 USD·Mg^–1^ of CO_2_ for the source-separated excreta. It is notable that at over 412 USD·Mg^–1^ of CO_2_ the source-separated scenario becomes less expensive than the mixed excreta scenario. In this analysis, we are only giving value to the carbon content. Source separation potentially could lead to higher quality biochar and may allow for a broader set of potential uses with higher economic value. Source separation could also produce a more consistent feedstock to pyrolysis, allowing for better optimization of operating conditions. Both feedstock and operating conditions have been shown to influence carbon stability.^82^ Producing biochar with constant properties and higher carbon stability may create greater economic opportunities, including carbon sequestration credits. Future studies may provide a more in-depth accounting of carbon and explore how biochar from fecal sludge compares to biochar from other feedstocks.

When tracking nitrogen, phosphorus, and potassium through the mixed excreta scenario, a fraction of the nutrients is unrecoverable due to pit latrines (Figure 4d). After pretreatment by dewatering, most of the remaining nutrients continue to the liquid treatment bed and are recoverable in a combined liquid. Specifically, this recovered liquid contains 27% [17–43%] of the nitrogen, 63% [50–76%] of the phosphorus, and 66% (58–73%) of the potassium. The biochar from the mixed excreta accounts for 7% [5–11%] of the potassium, 5% [2–13%] of the phosphorus, and <1% of the nitrogen. Source separation increases the potential for resource recovery with possibly higher value fertilizers (Figure 4e). For example, struvite is recovered, accounting for 40% [24–52%] of the phosphorus and 2% [1–4%] of the nitrogen. Ammonium sulfate is recovered, containing 59% [51–68%] of the nitrogen. After liquid bed treatment, the recovered liquid contains 70% [58–84%] of the potassium, 18% [11–24%] of the nitrogen, and 5% [3–6%] of the phosphorus. Additionally, more nutrients are captured in the biochar with source-separation, with 19% [9–35%] of the potassium, 14% [5–36%] of the phosphorus, and 18% [11–23%] of the nitrogen.

The recovered nutrients from the two scenarios present meaningful opportunities to offset per capita cost by the sale of the fertilizers. Assigning the nutrients’ monetary worth based on a percent of market value allows us to explore the potential to offset cost (Figure 4f). For the mixed excreta scenario, the reduction in cost is relatively nominal with 0.04 [0.03–0.09] USD·cap^–1^·d^–1^ when nutrients are 20% of market value and 0.03 [0.02–0.08] USD·cap^–1^·d^–1^ when nutrients are set to market value. The financial benefit of selling nutrients is greater for the source-separated scenario, shown by the steeper line for the per capita cost compared to the mixed excreta. When nutrients are 20% of market value, the per capita cost is 0.08 [0.06–0.13] USD·cap^–1^·d^–1^, and when nutrients are set to market value, the per capita cost is 0.04 [0.03–0.10] USD·cap^–1^·d^–1^. While the source-separated scenario allows more nutrients to be recovered, it also allows for those of higher value to be recovered (i.e., struvite and ammonium sulfate), leading to the observed financial benefit. The mixed excreta scenario recovers nutrients only as the combined liquid effluent from bed treatment. Although this liquid may not have comparable economic value to commercial fertilizers,^44^ it could provide a meaningful product, particularly in low-resource communities.^83^ Therefore, the potential value of the recovered resources may go beyond the offset in costs analyzed here.

While this study provides insights into the financial viability and environmental implications of fecal sludge management via OP technology, it has several limitations. In particular, the two deployment scenarios of the OP technology were compared to one benchmark system (i.e., pit latrines, transport, and anaerobic treatment); however, multiple pathways exist to provide safely managed sanitation (e.g., centralized versus decentralized) and should be considered before deployment of any system.^84,85^ Also, our analysis assumes that a maintenance network is in place to provide the necessary support to the OP. In accordance with ISO 31800 and ISO 30500, the routine maintenance of non-sewered sanitation systems and fecal sludge treatment units needs to be outlined, which can provide guidance on the development of this network.^86,87^ Furthermore, our technical assumptions use both laboratory-based experiments and field deployment of the Biogenic Refinery. The accuracy of these assumptions may vary, especially in different contexts over the entire lifetime of the OP. Long-term, continuous field studies may provide updated assumptions to further inform our analysis. Future studies also need to consider both social and stakeholder factors that will influence sustained adoption.^32^

This research provides insight into the deployment of a pyrolysis-based OP technology, while identifying potential barriers. For decision variables, the type of frontend (i.e., pit latrine or urine-diverting dry toilets) can contribute substantially to overall per capita costs and, in some cases, emissions. The average number of users per toilet informs deployment of the OP across a community. Although deciding the number of users per toilet is not feasible in most situations, aggregating toilets to a central collection tank (e.g., deployments at apartment buildings or neighborhoods) or public toilets (e.g., deployments at schools or parks) could be considered as it directly influences the economic and environmental sustainability of the OP in both deployment scenarios. For the treatment of mixed excreta, the greatest opportunities to lower per capita costs and GHG emissions include decreasing pit latrine emptying fees and having more frequent emptying periods, respectively. Source-separation through urine-diverting dry toilets creates opportunities for resource recovery that can potentially offset costs through carbon sequestration and recovery of nutrients. Integrating the produced heat from pyrolysis with the liquid treatment for nitrogen recovery could lower costs associated with ion exchange. Additionally, further studies on biochar quality may introduce greater opportunities to lower costs, especially for the treatment of mixed excreta and when supplementing additional feedstocks. Costs may also be offset from the sale of briquettes (produced from biochar) for cooking; however, emissions and health risks to users should be also considered for these practices.^85^ Contextual parameters such as diet (i.e., calorie intake) can impact the GHG emissions from the treatment of mixed excreta. Conversely, the protein intake of different populations provides distinctive opportunities for nutrient recovery from the treatment of source-separated excreta. Future studies may also investigate the impact of the context-specific toilets (including wet versus dry pit latrines) as well as cleansing practices (washing versus paper-based products). In contexts with varying population density, it may be important to optimize deployment by mapping transport routes. For technology parameters, further research into how the energy production from the Biogenic Refinery could be leveraged for pretreatment may further lower costs and provide a more sustainable system in locations where electricity blackouts are common.

Ultimately, this research reveals that thermal treatment (via pyrolysis) can be leveraged for low-cost, low-GHG, community-scale, non-sewered sanitation systems. Such treatment also allows opportunities for carbon sequestration and nutrient recovery. While technology deployment should consider a broad set of other contextual factors (e.g., stakeholder engagement and social acceptability),^30^ the economic and environmental feasibility of these systems shows promise. Overall, the relatively low cost and emissions from this pyrolysis-based OP technology demonstrate that it should be a part of the collection of fecal sludge treatment practices to eliminate global sanitation gaps moving forward.

## Supporting Information Available

Emissions estimates; two baseline scenarios for the Biogenic Refinery; baseline scenario costs and greenhouse emissions by percent; baseline scenarios broken down by unit processes; impact of significant parameters on costs and greenhouse emissions; daily per capita cost with supplemental addition of an agricultural residue; description of the Biogenic Refinery (defining the system); general approach to quantitative scenario modeling; addition of agricultural residues to treatment of mixed excreta; details on emptying and conveyance from pit latrines (PDF)
Inputs for unit processes in the analysis (XLSX)
