# Supplementary material for: Financial Viability and Environmental Sustainability of Fecal Sludge Treatment with Pyrolysis Omni Processors
Source: ACS Environ Au. 2022 Jul 29;2(5):455–66. doi: 10.1021/acsenvironau.2c00022 (PMC9502014; doi:10.1021/acsenvironau.2c00022)
Supplement: Supplementary file 1 — vg2c00022_si_001.pdf [file vg2c00022_si_001.pdf]

Supporting Information for

Financial viability and environmental sustainability of fecal sludge treatment with pyrolysis Omni Processors

Rowles, Lewis Stetson[1,2]*; Morgan, Victoria L.[1]; Li, Yalin[1]; Zhang, Xinyl[3]; Watabe, Shion[3]; Stephen, Tyler[3]; Lohman, Hannah A.C.[3]; DeSouza, Derek[4]; Hallowell, Jeff[4]; Cusick, Roland D.[3]; Guest, Jeremy S.[1,3]*

[1]Institute for Sustainability, Energy, and Environment, University of Illinois Urbana−Champaign, Urbana, Illinois 61801, United States

[2]Current Affiliation: Department of Civil Engineering and Construction, Georgia Southern University, Statesboro, Georgia 30458, United States

[3]Department of Civil & Environmental Engineering, University of Illinois Urbana−Champaign, Urbana, Illinois 61801, United States

[4]Biomass Controls PBC, Woodstock, Connecticut 06281, United States

**Corresponding authors: Lewis Stetson Rowles, email: lrowles@georgiasouthern.edu, phone: (912) 478-1894; Jeremy S. Guest, email: jsguest@illinois.edu, phone: (217) 244-9247*

Table of Contents (12 total pages):

Table S1. Emissions estimates

Table S2. Inputs for units used in the analysis

Figure S1. Two baseline scenarios for the Biogenic Refinery

Figure S2. Baseline scenarios costs and greenhouse emissions by percent

Figure S3. Baseline scenarios broken down by unit processes

Figure S4. Impact of significant parameters on costs and greenhouse emissions

Figure S5. Daily per capita cost with supplemental addition of an agricultural residue

Section S1. Description of the Biogenic Refinery (defining the system)

Section S2. General approach to quantitative scenario modeling

Section S3. Addition of agricultural residues to treatment of mixed excreta

Section S4. Details on emptying and conveyance from pit latrines

Supporting References

Table S1. Emissions estimates, based on relevant literature[1–14] and measured emissions for the Omni-Processor system.

Parameter	Expected Value	Minimum	Maximum	Distribution
Collected fecal sludge (pit latrines)				
Latrine sludge: Total volume [$L\cdot cap^{-1}\cdot yr^{-1}$]	270.0	-	-	(Constant)
Latrine sludge: Total solids [$g\cdot L^{-1}$]	25.0	15	48	Triangular
Latrine sludge: Emptying period [yr]	0.5	0.2	1	Triangular
Latrine sludge: N leached from latrine [%]	13.0	1	50	Uniform
Latrine sludge: P leached from latrine [%]	18.0	0	37	Uniform
Latrine sludge: K leached from latrine [%]	21.0	11	31	Uniform
Transportation				
Transportation [kg CO2eq$\cdot t^{-1}\cdot km^{-1}$]	0.194	0.0576	0.526	Triangular
Thermal drying				
Carbon dioxide emissions [% of total C]	1.6	0	3.2	Uniform
Methane emissions [% of total C]	0.03	0	0.06	Uniform
Ammonia emissions [% of total N]	0.26	0	0.52	Uniform
Transformation of NH_3 to N_2O [% of NH_3]	1	0	2	Uniform
Pyrolysis				
SO_2 emissions [mg/m^3][a]	-	38.61	85.88	Uniform
CO emissions [mg/m^3][a]	-	833.83	1119.39	Uniform
Hg emissions [mg/m^3][a]	-	0	0.01	Uniform
Cd emissions [mg/m^3][a]	-	0.01	0.03	Uniform
As emissions [mg/m^3][a]	-	0.01	0.02	Uniform
Dioxin and furans emissions [ng/m^3][a]	-	0.24	0.4	Uniform
Methane emissions [% of total C] [b]	0	-	-	-
N_2O emissions [% of total N]	1.5	0.07	2.95	Uniform
C released as gaseous products [% of total C][a]	60	50	70	Uniform
N released as gaseous products [% of total N][a]	80	70	90	Uniform
K released as gaseous products [% of total K][a]	25	10	40	Uniform
P released as gaseous products [% of total P][a]	65	50	80	Uniform
NH_3 emissions [% of total N]	3	1	4	Triangular
HNCO emissions [% of total N]	3	0.5	10	Triangular
Transformation of HNCO to NH_3 [% of HNCO]	50	25	75	Uniform

[a] This data was provided by Biomass Controls.

[b] Pyrolysis is assumed to produce emissions that are similar to combustion, with any methane from the reactor's pyrolysis zone being oxidized during catalysis.

Table S2. Inputs for units used in the analysis. This information is from the code is that is openly available on Github.[15]
(Table S2 is included as an Excel spreadsheet:
Rowles_et_al_unit_inputs_Table_S2.xlsx)

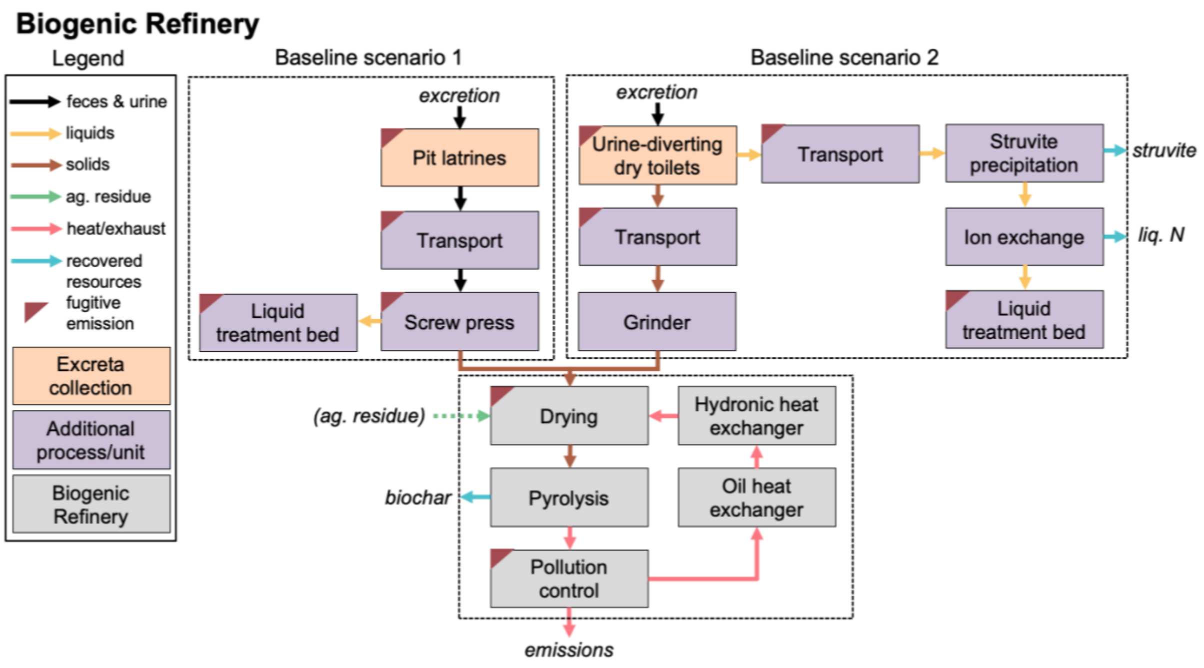

Figure S1. Two baseline scenarios for the Biogenic Refinery evaluated, including pit latrines and container-based sanitation. Various subsets of these scenarios were evaluated across the simulation space of decision variables, contextual parameters, and technological parameters.

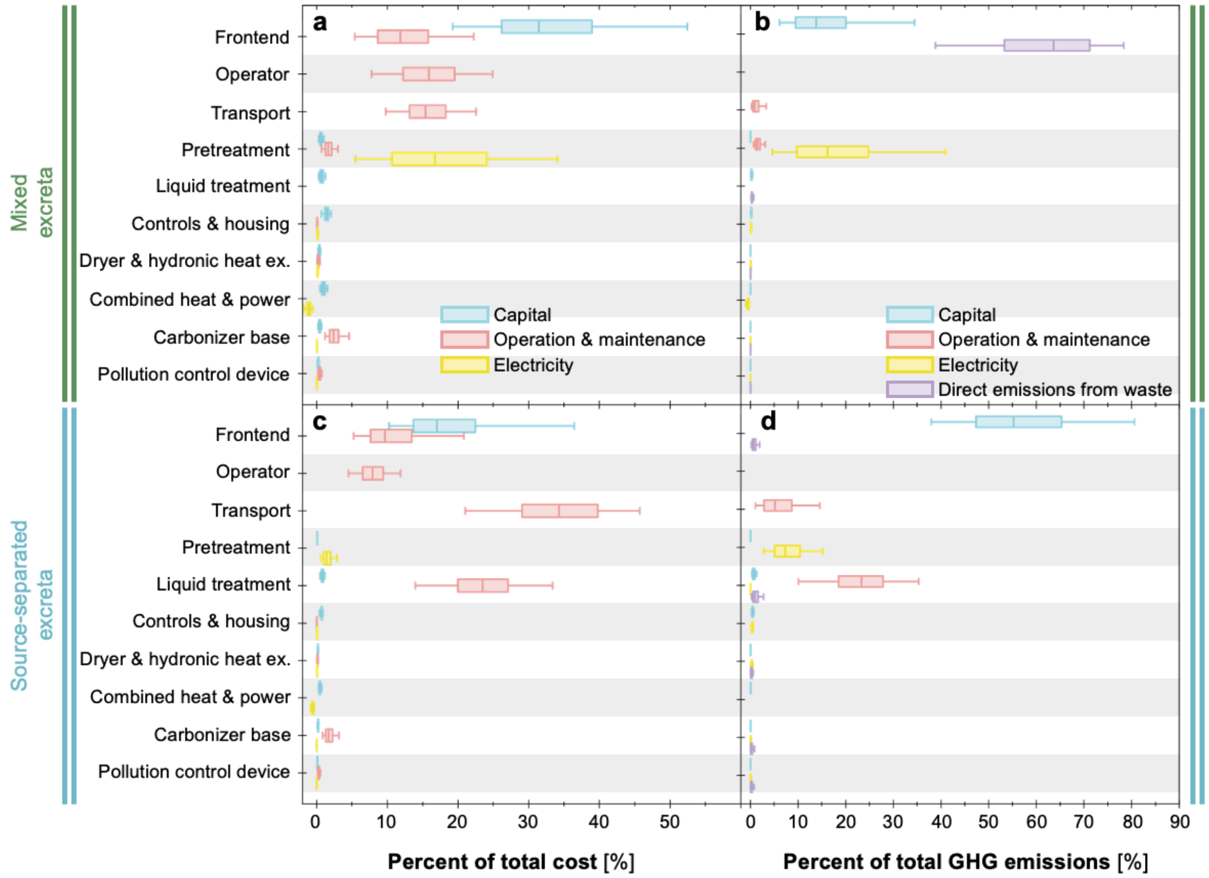

Figure S2. Baseline scenarios broken down by unit processes for the Biogenic Refinery. Percent of total daily per capita cost is shown for treatment of mixed excreta (a) and source separated excreta (c). Percent of annual GHG emissions per user is shown treatment of mixed excreta (b) and source separated excreta (d). This analysis assumes 12,000 users, a 20-year lifetime, and 10,000 units produced at scale. The top half of each graph shows results for the treatment of mixed excreta from pit latrines, and the bottom half of each graph shows results for the treatment of source-separated excreta from urine-diverting dry toilets. The left panels show the breakdown of costs with the relative contributions of capital, general O&M, electricity, and sale of recovered resources to the median per capita cost. The right panels show the breakdown of emissions per capita per year with the relative contributions from capital, operation and maintenance, energy, and direct emissions to the median GHG emissions. The box and whisker plots represent the median values (center line), 25th and 75th percentiles (bottom and top of box), 10th and 90th percentiles (lower and upper whiskers), and 5th and 5th percentiles (points on either end of the whiskers) from the uncertainty analysis with 10,000 Monte Carlo simulations.

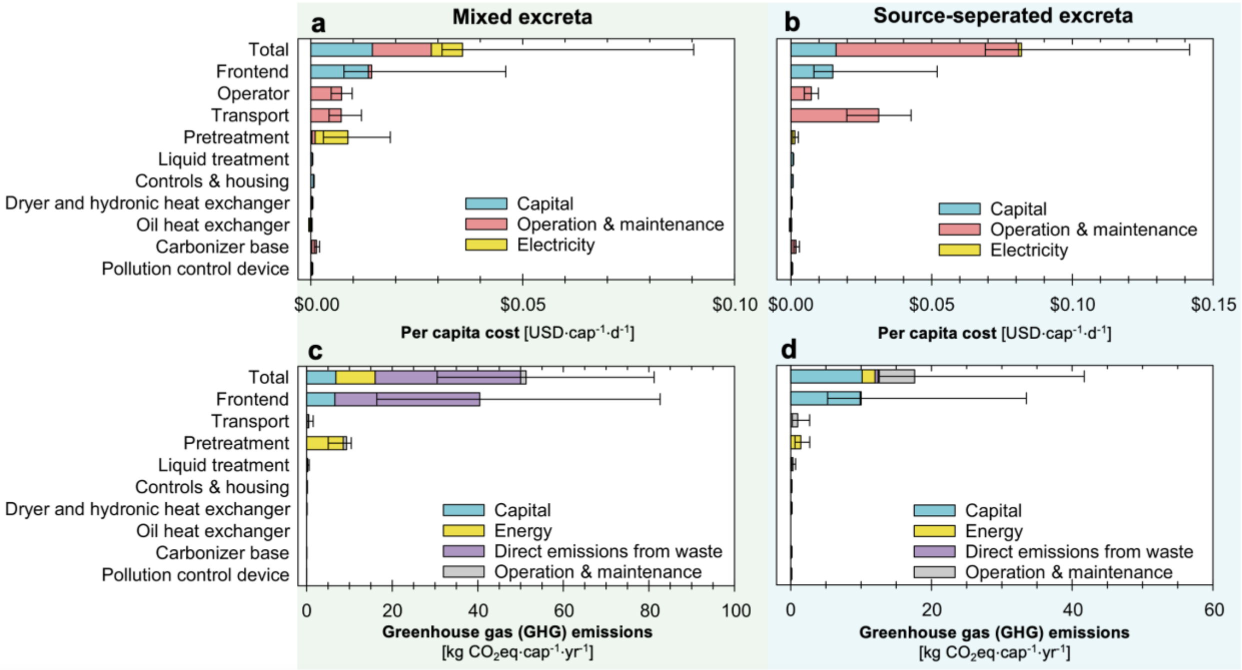

Figure S3. Baseline scenarios broken down by unit processes for the Biogenic Refinery. Daily per capita cost is shown for treatment of mixed excreta (a) and source separated excreta (c). Annual GHG emissions per user is shown for treatment of mixed excreta (b) and source separated excreta (d). This analysis assumes 12,000 users, a 20-year lifetime, and 10,000 units produced at scale. The top half of each graph shows results for the treatment of mixed excreta from pit latrines (*baseline scenario 1* from Figure S1), and the bottom half of each graph shows results for the treatment of source-separated excreta from urine-diverting dry toilets (*baseline scenario 2* from Figure S1). The left panel shows breakdown of daily per capita costs with the relative contributions of capital, general O&M, electricity, and sale of recovered resources to the median per capita cost. The right panel shows the breakdown of emissions per capita per year with the relative contributions from capital, energy, direct emissions, and operation and maintenance to the median GHG emissions. Error bars show the 5th and 95th percentile values from the uncertainty analysis.

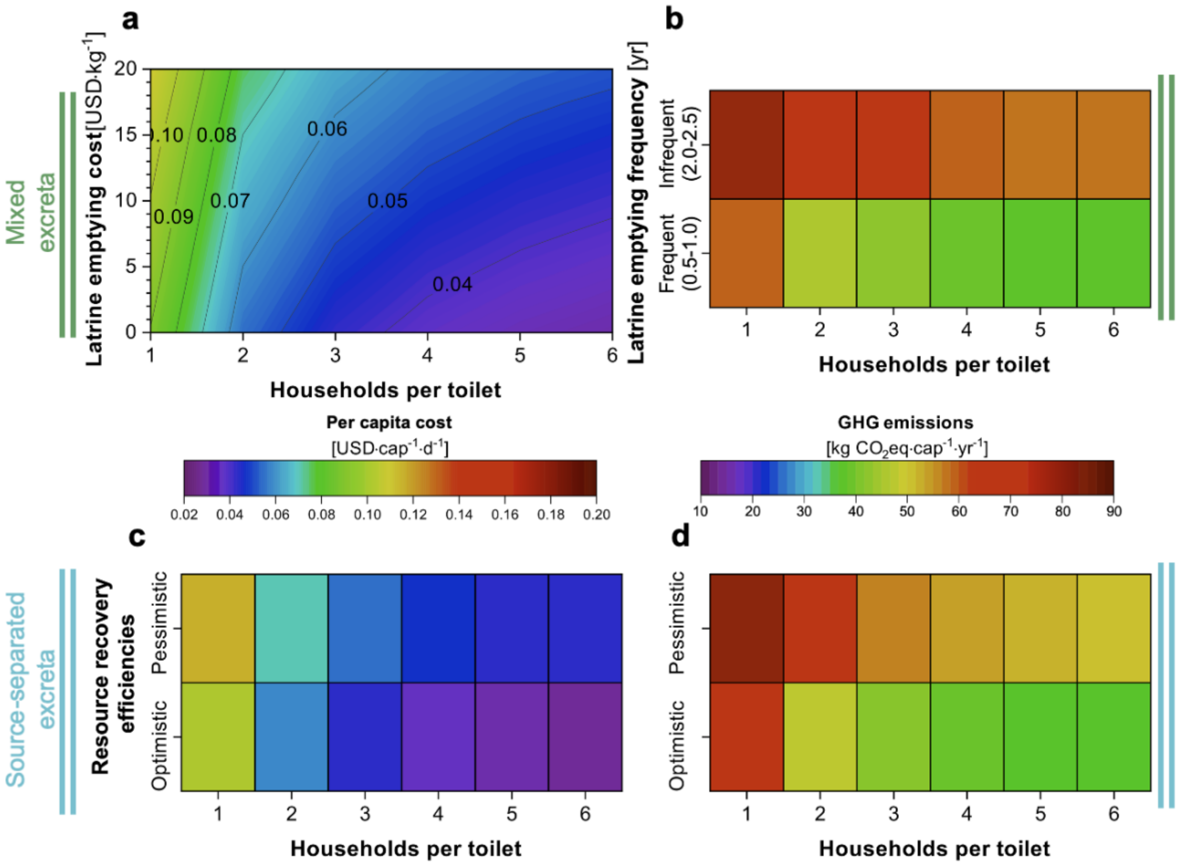

Figure S4. Daily per capita cost from treatment of a mixed excreta stream by the Biogenic Refinery varying households per toilet and latrine emptying fee (a). Greenhouse gas (GHG) emissions from treatment of a mixed excreta stream by the Biogenic Refinery varying households per toilet and latrine emptying frequency (b). Daily per capita cost from treatment of a source-separated excreta stream by the Biogenic Refinery varying households per toilet and resource recovery efficiencies (c). GHG emissions from treatment of a source-separated excreta stream by the Biogenic Refinery varying households per toilet and resource recovery efficiencies (d).

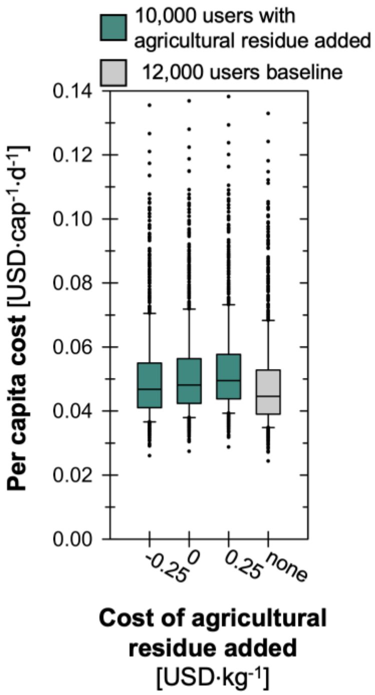

Figure S5. Daily per capita cost when addition of an agricultural residue is considered to supplement feedstock for a system serving 10,000 users for treatment of mixed excreta. The boxplots represent the median values (center line), 25th and 75th percentiles (bottom and top of box), 10th and 90th percentiles (lower and upper whiskers), and outliers from the uncertainty analysis.

Section S1. Description of the Biogenic Refinery

The Carbonizer Base is the central location for the combined pyrolysis and combustion process. The feedstock is received into the pyrolysis pot in the Carbonizer Base, where it is flash pyrolyzed releasing volatile gases into the Pollution Control Device. These gases are then pulled through a noble metal coated catalyst that uses a thermochemical process causing combustion of the Volatile Organic Compounds, Carbon Monoxide and particulate matter. Due to the combustion of gases, the Pollution Control Devise is the hottest location of the refinery ranging between 550-700 ºC. This range is closely monitored as pyrolysis usually starts at 300 deg C and all of the volatile gases are released at 550 ºC. This temperature is also important when confirming treatment of the fecal sludge, as it serves as our evidence for inactivation of the microbes in the sludge. After the gases are combusted, the exhaust travels below a baffle plate to encourage the fallout of particulates before it proceeds to the heat exchanger.

The PCD is the middle section of the Biogenic Refinery and its primary responsibilities include pollution control and production of thermal energy for drying of the feedstock. Due to the inefficiencies of the pyrolysis process, there are typically pollutants in the exhaust. In order to treat these pollutants, the Biogenic Refinery has a catalyst, similar to a catalytic converter in a car, to ensure destruction of the pollutants before they can be released into the surrounding environment. The process of destroying the pollutants requires the catalyst to maintain temperatures above 315 ºC, and additional energy is released during this process. The temperature of the catalyst is closely monitored because the catalyst wash coat will start to degrade above 800 ºC.

The final section of the Biogenic Refinery is the Heat Exchanger (HX). Biomass Controls offers three types of heat exchanger methods, a Forced Air Heat Exchanger (FAHX), a Hydronic Heat Exchanger (HHX), and a Combined Heat and Power (CHP) system. The purpose of the heat exchanger is to utilize the thermal energy that is created in the pyrolysis process while ensuring that the stack maintains temperatures above 110 deg C to prevent condensation from lining the stack wall. Creosote is a carbonaceous material that is formed during pyrolysis.

In the FAHX system, there is an exchange between the exhaust gas and ambient air to use the thermal energy to heat a nearby space. These types of systems are typically used in cold climates as it does not require any water that would otherwise freeze, and the heat can be used to heat a cabin for the operator.

The HHX system is used for applications that require drying of the feedstock before the refinery is capable of processing the material. The heat is exchanged between the exhaust gas and water, which is then pumped into radiators connected to a dryer. The refinery monitors the

temperature of the water to ensure that the feedstock is being sufficiently dried before entering the refinery.

The CHP system is used to generate additional electricity that the refinery and/or facility can use to decrease the unit's electrical demand on the electrical grid. This type of system is required for ISO 31800 certification as the treatment unit needs to be energy independent when processing fecal sludge.

Section S2. General approach to quantitative scenario modeling

Section S2.1. Economic analysis. For the Biogenic Refinery, we calculated the total system material cost using the bill of materials. The design team provided a bill of materials that included several of the main components, their weight, and material composition. A more detailed bill of materials was developed using the system's user manual and related patents. Each part was included in its respective unit process for the estimates of capital costs. Specifically, initial capital costs are distributed over the lifetime of the system (20 years), with a discount rate (2-8%) adjusting for the diminishing value of money over time. To provide a conservative estimate of per capita costs associated with serial production of each system at scale, we used a generalized learning curve function[16] to estimate the capital cost of the 10,000th unit. Operation and maintenance (O&M) costs included ongoing costs for materials and labor. Assumptions for O&M were developed from a detailed maintenance activity schedule to estimate the need for replacement parts, along with costs of labor wages associated with the different levels of skills required for maintenance (e.g., service team, electrician, Biomass Controls, etc.). Finally, costs from electricity requirements were estimated based on the energy needs of specific components and parts, along with typical electricity costs per kilowatt-hour. For the energy requirements of specific components and parts, data from a published study on the system.[17]

Section S2.1. Environmental analysis. Capital impacts were calculated using the bill of materials and vendor websites to identify materials and processes associated with each. We then compiled an inventory of emissions associated with each material and process or used similar items as surrogates when necessary. We used global emissions values for each item. Emissions were then converted to unit global warming potential (e.g., kg of CO_2 equivalent per kilogram of material). Impacts associated with electricity were calculated from the OP's energy requirements and the unit environmental impacts associated with grid electricity.

For environmental impacts originating directly from the excreta, we estimated direct GHG emissions, which include methane and N_2O released from the degradation of bodily waste. By mass, methane from biogenic sources is estimated to contribute 28 times the climate change impact of non-biogenic carbon dioxide, while N_2O has an impact 265 times that of carbon dioxide.[18] Total CH_4 and N_2O emissions were multiplied by these factors to represent all direct emissions as an equivalent mass of carbon dioxide. Methane and N_2O released from bodily waste in sanitation systems depend on the environmental conditions present and the treatment processes being employed, and they are directly related to the quantities of carbon (assumed to be proportional to COD[19]) and nitrogen excreted in bodily waste. We used general ranges of COD

and nitrogen excretion found in the literature (Table S1).[20] In our process models, the specific configurations determine what fraction of excreted carbon and nitrogen enter certain treatment processes. The analysis focused on emissions that may occur during active treatment processes (e.g., thermal drying, pyrolysis) with expected emissions based on data from relevant literature (Table S1). While carbon dioxide emissions contribute no global warming impacts (as they are biogenic), they are incorporated to track carbon losses throughout each system. Thermal drying processes are expected to release minimal quantities of methane and ammonia (a fraction of which may transform to N_2O in the atmosphere).[1,2] Pyrolysis of bodily waste is assumed to release N_2O of up to approximately 3% of total nitrogen.[2] Other emissions that were documented in a field study for OP were also included in our analysis. These included SO_2, CO, Hg, Cd, As, and dioxin and furans, all of which were estimated to be emitted at the exhaust flow rate of the system.

Using the maintenance activity schedule and consumables from system processes, the impacts due to O&M of the OP were estimated. The CO_2 emissions associated with transportation were estimated based on transport distances and the volume transported.[21]

Section S3. Addition of agricultural residues to treatment of mixed excreta.

The addition of an agricultural residue is considered for the treatment of mixed excreta with 10,000 users. Details on this analysis are shown in the _models.py file as (Scenario C or sysC). The mass of residue added is equivalent to achieving the same mass loading rate to the Carbonizer Base as the scenario with 12,000 users (i.e., the mass loading rate into pyrolysis is the same for scenario with 12,000 users as the scenario with 10,000 users plus agricultural residue). For this system, rice husks were supplemented as the agricultural residue with a typical caloric value of 14.693 $MJ \cdot kg^{-1}$, moisture content of 8.47 %, C content of 38.5%, and N content of 0.45% from the literature.[22]

Section S4 Details on emptying and conveyance from pit latrines

Baseline assumptions related to emptying and conveyance were adopted from Trimmer et al. 2020.[23] Details on these assumptions and probability density distributions are shown in the _trucking section of Table S2 and the Python files (_trucking.py, _systems.py, and _models.py). The conveyance of the sludge first requires pumping the sludge from pit latrines to a tanker truck. This truck then transports the collected sludge to a central facility where it is subsequently treated by the pyrolysis Omni Processor. Our analysis included interdependences between emptying period, trucking interval, waste density and volume, and emptying fee. First, trucking interval was defined as the emptying period (i.e., time interval between trips). Next, the truck load (mass per load) was based on the sludge mass from the toilets, trucking interval, and number of toilets. The transportation fee (transportation fee per trip) was then calculated considering truck load and sludge density (to convert to volume of sludge) using a power law regression (as shown in the code on Github[15]).

Supporting References

(1) Deng, W.-Y.; Yan, J.-H.; Li, X.-D.; Wang, F.; Zhu, X.-W.; Lu, S.-Y.; Cen, K.-F. Emission Characteristics of Volatile Compounds during Sludges Drying Process. *J. Hazard. Mater.* **2009**, *162* (1), 186–192. https://doi.org/10.1016/j.jhazmat.2008.05.022.

(2) Barton, P. K.; Atwater, J. W. Nitrous Oxide Emissions and the Anthropogenic Nitrogen in Wastewater and Solid Waste. *J. Environ. Eng.* **2002**, *128* (2), 137–150. https://doi.org/10.1061/(ASCE)0733-9372(2002)128:2(137).

(3) Shao, J.; Yan, R.; Chen, H.; Wang, B.; Lee, D. H.; Liang, D. T. Pyrolysis Characteristics and Kinetics of Sewage Sludge by Thermogravimetry Fourier Transform Infrared Analysis. *Energy Fuels* **2008**, *22* (1), 38–45. https://doi.org/10.1021/ef700287p.

(4) Li, Y.; Leow, S.; C. Fedders, A.; K. Sharma, B.; S. Guest, J.; J. Strathmann, T. Quantitative Multiphase Model for Hydrothermal Liquefaction of Algal Biomass. *Green Chem.* **2017**, *19* (4), 1163–1174. https://doi.org/10.1039/C6GC03294J.

(5) IPCC. *2019 Refinement to the 2006 IPCC Guidelines for National Greenhouse Gas Inventories, Volume 4: Agriculture, Forestry and Other Land Use*; National Greenhouse Gas inventories Programme, Eggleston H.S., Buendia L., Miwa K., Ngara T. and Tanabe K. (eds): Japan, 2019.

(6) IPCC. *2019 Refinement to the 2006 IPCC Guidelines for National Greenhouse Gas Inventories, Volume 5: Waste*; National Greenhouse Gas inventories Programme, Eggleston H.S., Buendia L., Miwa K., Ngara T. and Tanabe K. (eds): Japan, 2019.

(7) Strande, L.; Schoebitz, L.; Bischoff, F.; Ddiba, D.; Okello, F.; Englund, M.; Ward, B. J.; Niwagaba, C. B. Methods to Reliably Estimate Faecal Sludge Quantities and Qualities for the Design of Treatment Technologies and Management Solutions. *J. Environ. Manage.* **2018**, *223*, 898–907. https://doi.org/10.1016/j.jenvman.2018.06.100.

(8) Orner, K. D.; Mihelcic, J. R. A Review of Sanitation Technologies to Achieve Multiple Sustainable Development Goals That Promote Resource Recovery. *Environ. Sci. Water Res. Technol.* **2018**, *4* (1), 16–32. https://doi.org/10.1039/C7EW00195A.

(9) Jacks, G.; Sefe, F.; Carling, M.; Hammar, M.; Letsamao, P. Tentative Nitrogen Budget for Pit Latrines – Eastern Botswana. *Environ. Geol.* **1999**, *38* (3), 199–203. https://doi.org/10.1007/s002540050415.

(10) Lagerstedt, E.; Jacks, G.; Sefe, F. Nitrate in Groundwater and N Circulation in Eastern Botswana. *Environ. Geol.* **1994**, *23* (1), 60–64. https://doi.org/10.1007/BF00773140.

(11) Nyenje, P. M.; Foppen, J. W.; Kulabako, R.; Muwanga, A.; Uhlenbrook, S. Nutrient Pollution in Shallow Aquifers Underlying Pit Latrines and Domestic Solid Waste Dumps in Urban Slums. *J. Environ. Manage.* **2013**, *122* (Supplement C), 15–24. https://doi.org/10.1016/j.jenvman.2013.02.040.

(12) Phillips, I.; Burton, E. Nutrient Leaching in Undisturbed Cores of an Acidic Sandy Podosol Following Simultaneous Potassium Chloride and Di-Ammonium Phosphate Application. *Nutr. Cycl. Agroecosystems* **2005**, *73* (1), 1–14. https://doi.org/10.1007/s10705-005-6080-8.

(13) Muff, J. Chapter 3 - Electrochemical Oxidation – A Versatile Technique for Aqueous Organic Contaminant Degradation. In *Chemistry of Advanced Environmental Purification Processes of Water*; Søgaard, E. G., Ed.; Elsevier: Amsterdam, 2014; pp 75–134. https://doi.org/10.1016/B978-0-444-53178-0.00003-1.

(14) Samolada, M. C.; Zabaniotou, A. A. Comparative Assessment of Municipal Sewage Sludge Incineration, Gasification and Pyrolysis for a Sustainable Sludge-to-Energy Management in Greece. *Waste Manag.* **2014**, *34* (2), 411–420. https://doi.org/10.1016/j.wasman.2013.11.003.

(15) *QSDsan: Developed Systems*. https://qsdsan.readthedocs.io/en/latest/Developed_Systems.html (accessed 2022-07-02).

(16) Wong, L. F. A Generalized Learning Curve Adapted for Purchasing and Cost Reduction Negotiations. *Adv. Oper. Res.* **2013**, *2013*, 1–9. https://doi.org/10.1155/2013/584762.
(17) Myers, T.; Schoebitz, L.; Woolley, S.; Sanchez Ferragut, J.; Thostenson, J.; Jooss, K.; Piascik, J.; Frechette, A.; Hotz, N.; Stoner, B. R.; Hallowell, J. Towards an Off-Grid Fecal Sludge Treatment Unit: Demonstrating Energy Positive Thermal Treatment. *Gates Open Res.* **2019**, *3*. https://doi.org/10.12688/gatesopenres.12929.1.
(18) Myhre, G.; Shindell, D.; Bréon, F.-M.; Collins, W.; Fuglestvedt, J.; Huang, J.; Koch, D.; Lamarque, J.-F.; Lee, D.; Mendoza, B.; Nakajima, T.; Robock, A.; Stephens, G.; Takemura, T.; Zhang, H. Anthropogenic and Natural Radiative Forcing. In *Climate Change 2013: The Physical Science Basis. Contribution of Working Group I to the Fifth Assessment Report of the Intergovernmental Panel on Climate Change*; Stocker, T. F., Qin, D., Plattner, G.-K., Tignor, M., Allen, S. K., Boschung, J., Nauels, A., Xia, Y., Bex, V., Midgley, P. M., Eds.; Cambridge University Press: Cambridge, United Kingdom and New York, NY, USA, 2013.
(19) Tchobanoglous, G.; Stensel, H. D.; Tsuchihashi, R.; Burton, F.; Abu-Orf, M.; Bowden, G.; Pfrang, W. *Wastewater Engineering: Treatment and Resource Recovery*, 5th ed.; Metcalf & Eddy, Inc., AECOM, McGraw-Hill: New York, 2014.
(20) Rose, C.; Parker, A.; Jefferson, B.; Cartmell, E. The Characterization of Feces and Urine: A Review of the Literature to Inform Advanced Treatment Technology. *Crit. Rev. Environ. Sci. Technol.* **2015**, *45* (17), 1827–1879. https://doi.org/10.1080/10643389.2014.1000761.
(21) Trimmer, J. T.; Byrne, D. M.; Lohman, H. A. C.; Guest, J. S. Preliminary Greenhouse Gas (GHG) Emissions Analysis of Four Gates Sanitation Systems: Emissions During Steady-State Operation. *Gates Open Res* **2020**, *4*. https://doi.org/10.21955/gatesopenres.1116564.1.
(22) Channiwala, S. A.; Parikh, P. P. A Unified Correlation for Estimating HHV of Solid, Liquid and Gaseous Fuels. *Fuel* **2002**, *81* (8), 1051–1063. https://doi.org/10.1016/S0016-2361(01)00131-4.
(23) Trimmer, J. T.; Lohman, H. A. C.; Byrne, D. M.; Houser, S. A.; Jjuuko, F.; Katende, D.; Banadda, N.; Zerai, A.; Miller, D. C.; Guest, J. S. Navigating Multidimensional Social–Ecological System Trade-Offs across Sanitation Alternatives in an Urban Informal Settlement. *Environ. Sci. Technol.* **2020**, *54* (19), 12641–12653. https://doi.org/10.1021/acs.est.0c03296.
